# Individual and joint trajectories of change in bone, lean mass and physical performance in older men

**DOI:** 10.1186/s12877-020-01560-5

**Published:** 2020-05-05

**Authors:** Peggy M. Cawthon, Neeta Parimi, Lisa Langsetmo, Jane A. Cauley, Kristine E. Ensrud, Steven R. Cummings, Nancy E. Lane, Andrew R. Hoffman, Jodi Lapidus, Thomas M. Gill, Charles E. McCulloch, Marcia L. Stefanick, Deborah M. Kado, Rebecca Drieling, Eric S. Orwoll

**Affiliations:** 1 California Pacific Medical Center, Research Institute, San Francisco, CA USA; 2 Department of Epidemiology and Biostatistics, University of California, San Francisco Coordinating Center, 550 16th Street, 2nd floor, Box #0560, San Francisco, CA 94143 USA; 3 Division of Epidemiology and Community Health, University of Minnesota, Minneapolis, MN USA; 4 Department of Epidemiology, Graduate School of Public Health, University of Pittsburgh, Pittsburgh, USA; 5 Center for Chronic Disease Outcomes Research, Minneapolis VA Health Care System, Minneapolis, USA; 6 Department of Medicine, University of Minnesota, Minneapolis, MN USA; 7 University of California, Davis, CA USA; 8 Stanford University, Stanford, CA USA; 9 Oregon Health and Science University, Portland, OR USA; 10 Yale School of Medicine, New Haven, CT USA; 11 University of California, San Diego, CA USA

**Keywords:** Trajectories, Aging, Older men, Grip strength

## Abstract

**Background:**

Declines in bone, muscle and physical performance are associated with adverse health outcomes in older adults. However, few studies have described concurrent age-related patterns of change in these factors. The purpose of this study was to characterize change in four properties of muscle, physical performance, and bone in a prospective cohort study of older men.

**Methods:**

Using repeated longitudinal data from up to four visits across 6.9 years from up to 4681 men (mean age at baseline 72.7 yrs. ±5.3) participating in the Osteoporotic Fractures in Men (MrOS) Study, we used group-based trajectory models (PROC TRAJ in SAS) to identify age-related patterns of change in four properties of muscle, physical performance, and bone: total hip bone mineral (BMD) density (g/m^2^) and appendicular lean mass/ht^2^ (kg/m^2^), by DXA; grip strength (kg), by hand dynamometry; and walking speed (m/s), by usual walking pace over 6 m. We also described joint trajectories in all pair-wise combinations of these measures. Mean posterior probabilities of placement in each trajectory (or joint membership in latent groups) were used to assess internal reliability of the model. The number of trajectories for each individual factor was limited to three, to ensure that the pair-wise determination of joint trajectories would yield a tractable number of groups as well as model fit considerations.

**Results:**

The patterns of change identified were generally similar for all measures, with three district groups declining over time at roughly similar rates; joint trajectories revealed similar patterns with no cross-over or convergence between groups. Mean posterior probabilities for all trajectories were similar and consistently above 0.8 indicating reasonable model fit to the data.

**Conclusions:**

Our description of trajectories of change with age in bone mineral density, grip strength, walking speed and appendicular lean mass found that groups identified by these methods appeared to have little crossover or convergence of change with age, even when considering joint trajectories of change in these factors.

## Background

Poor physical performance is a harbinger of mortality [1], and it is strongly associated with age-related morbidity, including mobility limitations, falls, fractures, hospitalization, dependency, and long-term care [2–9]. In addition, osteoporosis and sarcopenia are important contributors to age-related declines in performance. Low bone mass can result in fractures, and in turn to both short- and long-term disability and functional dependence [10–12]. Sarcopenia (age-related muscle loss that is accompanied by reduced strength and physical performance) [13] Sarcopenia (age-disability and dependence. Although change in bone, muscle and physical performance may be associated with unfavorable outcomes (such falls, fractures, mortality and disability) [14–17], few studies have investigated the process of change in these factors concurrently.

In older men, we have described distinct trajectories of bone loss with age, demonstrating that the character of change is important, and that declines increases fracture risk and mortality [2, 14]. However, we have not extensively characterized change in muscle and physical performance. There is a gap in the literature with regard to characterization of change in several musculoskeletal factors concurrently: few, if any, papers have been published in this area. Studies that have reported change in measures simultaneously tend to report change in just two factors rather than a set of musculoskeletal variables [18, 19]. Muscle and bone are both generated from cells differentiated from mesenchymal stem cells, and as a result have overlapping genetic and biological underpinnings [20], that represents a closely linked “complex” in both biology and function. Thus, our ultimate goal is to test the hypothesis that when age-related deterioration in bone, muscle and physical performance occur in parallel their combined effects magnify the risk of poor functional and health outcomes among community-dwelling older men.

To quantify the trajectories, grip strength and walking speed were included for the physical performance/strength domain, because these measures are included in most of the consensus definitions of sarcopenia [21–23], and each have been consistently associated with poor outcomes such as mortality and disability in older adults [24, 25]. To quantify change in muscle, we used an approximation of muscle mass, appendicular lean body standardized to height squared (ALM/ht^2^) [26]. Total hip BMD was used as the measure of bone mineral density.

This report identifies joint and individual trajectory patterns in these musculoskeletal factors using data from the Osteoporotic Fractures in Men (MrOS) study, a prospective cohort study of older, community-dwelling men in order to provide a descriptive summary of how these factors concurrently change with age. We expected that we would identify subsets of men who had markedly rapid declines in these musculoskeletal factors, both individually and in combination.

## Methods

The MrOS Study has been previously described [27, 28]. Briefly, between March 2000 and April 2002, 5994 community-dwelling, ambulatory men age ≥ 65 years free from bilateral hip replacement were recruited to participate at six United States clinical centers (Birmingham, Alabama; Minneapolis, Minnesota; Palo Alto, California; Monongahela Valley near Pittsburgh, Pennsylvania; Portland, Oregon; and San Diego, California). All surviving participants were invited to return to the clinic for the Year 5 visit (Visit 2, March 2005–May 2006; N at visit, 5229) and the Year 7 visit (Visit 3, March 2007–March 2009; N at visit, 4681). A subset of participants were invited to an ancillary Year 3.5 Visit (Sleep Visit, December 2003–March 2005) designed to understand sleep habits of older men (N at visit =3153). Institutional review boards at all clinic centers and the San Francisco Coordinating Center (University of California, San Francisco and California Pacific Medical Center Research Institute) approved this study, and all men provided written informed consent at each study visit. Protocols were standardized across all visits unless otherwise noted; at each contact, all measures were completed during a single visit on the same day.

### Walking speed and grip strength

Physical performance was assessed at baseline and the Years 3.5, 5 and 7 visits [9]. Walking speed (m/s) was taken as the average of two trials over six meters at the participant’s usual pace. Grip strength was measured using Jamar dynamometers (Sammons Preston Rolyan, Bolingbrook, IL, USA), and the maximum effort from two trials of both hands was analyzed [29].

### Lean mass and bone mineral density

Total hip bone mineral density (BMD) and appendicular lean mass (ALM) were measured using Hologic 4500 dual energy x-ray absorptiometry (DXA) machines; the maximum percent difference between scanners was 1.2%. DXA scans were analyzed at each clinical center, with a centralized review of a random subset of scans and all problematic scans identified by technicians at the clinics. The primary measure of lean mass was ALM/ht^2^ (kg/m^2^).

### Other measures

Height was measured on wall-mounted Harpenden stadiometers (Holtain Ltd., Dyved, United Kingdom) and weight on balance beam or digital scales with standardized protocols. Body mass index (BMI) was calculated as weight (kg) / height^2^ (m^2^). Activity level was determined from the Physical Activity Scale for the Elderly (PASE) [30], a unit less measure where higher scores indicated a higher activity level. Self-rated health was classified as excellent/good (compared to fair/poor/very poor). Medical conditions included self-report of a physician’s diagnosis of the following: diabetes, stroke, myocardial infarction, non-skin cancer, high blood pressure, congestive heart failure, and Parkinson’s disease and were analyzed as the presence of one or more (vs. none). Functional limitations were assessed by self-report of at least some difficulty with any of the following tasks: walking 2–3 blocks, climbing stairs, shopping, cooking meals and heavy housework.

### Analysis subset

Men were included in the analyses if they had values for the main musculoskeletal measurements (walking speed, grip strength, ALM/ht^2^, and total hip BMD) at baseline and the Year 7 visit (Visit 3). We restricted the sample to this population because the ultimate goal is to determine whether trajectories up to the Year 7 predict outcomes subsequent to that visit. When available, Year 3.5 and Year 5 data was also used to determine the trajectories. Of the 5994 men at baseline, 1313 did not attend any part of the Year 7 visit: 1043 died before the visit; 101 were living but declined participation and 169 terminated participation in the study before the visit. At Year 7, 4681 men provided at least some data (including those who were classified as unable) for grip strength, walking speed, total hip BMD or ALM/ht^2^.

### Modeling of trajectories

First, these analyses described trajectories of each of four factors individually: grip strength, ALM/ht^2^, total hip BMD, and walking speed.

Second, we describe the joint trajectories in all pair-wise combinations of these factors (that is, concurrent change in grip strength-ALM/ht^2^; grip strength-total hip BMD; grip strength-walking speed; ALM/ht^2^-total hip BMD; ALM/ht^2^-walking speed; and total hip BMD-walking speed.)

We used group based trajectory modeling (GBTM) to identify patterns of change in these factors with the TRAJ procedure in SAS which identifies clusters of individuals following similar progressions of a specified phenotype over some measure of time (in our case, age) [31, 32].

A priori, we decided to limit the number of trajectories for each individual factor to three, to ensure that the pair-wise determination of joint trajectories would yield a tractable number of groups. Thus, using 3 trajectories for two individual factors results in 9 joint trajectories.

An individual was assigned to a group based on his highest posterior group probability over the change period. Using the final model as described above, we calculated mean posterior probabilities of placement in each trajectory (i.e., joint membership in latent groups) to assess internal reliability of the model. Patterns of change for each individual and joint membership in latent groups were depicted graphically. We tested whether characteristics of participants differed by the joint trajectory membership assigned, using ANOVA for normal continuous variables, Kruskal-Wallis for skewed continuous variables, and chi-square tests for categorical variables. Change in each factor was plotted graphically using a spline plot for the actual value of the factor from the single trajectory model for each age group. Similarly for joint trajectories, the actual value for each age and joint trajectory was calculated and plotted using a spline fit after group assignment was determined [33]. Although the determination of trajectories was determined jointly for each pairwise comparison (e.g. grip strength and BMD together), we show graphically trajectories of grip strength by category of BMD trajectory (and vice versa). A three-way graphical description of the data (grip trajectory vs BMD trajectory vs age) was too unwieldly.

### Missing data or unable to complete a measure

Men who did not have values for the performance-based tests (walking speed and grip strength) at any visit because they attempted but were considered unable to do the measure for physical reasons (per study staff or personal assessment) were not included in the trajectories analyses. These men were included as a separate group in the characteristics table. Men who had missing values for the performance measures or the DXA measures for other reasons (for example, “questionnaire only” participants without a clinic visit) were included in the trajectories analyses with the appropriate value set to missing. Of the 4681 men at the Year 5 visit, 259 men were unable to complete the grip strength exam at all visits; 18 were unable to complete the walking test at all visits. In addition, one had missing total hip BMD at all visits, and 14 were missing ALM/ht^2^ data at all visits. Thus, the N for each classification of trajectories in each measure was 4681 for grip strength (4422 with a trajectory calculated and 259 unable to complete at all visits who were included as a separate group); 4680 for walking speed (includes 4662 with a trajectory calculated and 18 who were unable to complete at all visits who were included as a separate group); 4680 for total hip BMD and 4667 for ALM/ht^2^. Men missing values at all visits were excluded from the individual and pair-wise joint trajectories as appropriate. (Additional file 1).

## Results

**Fig. 1:**
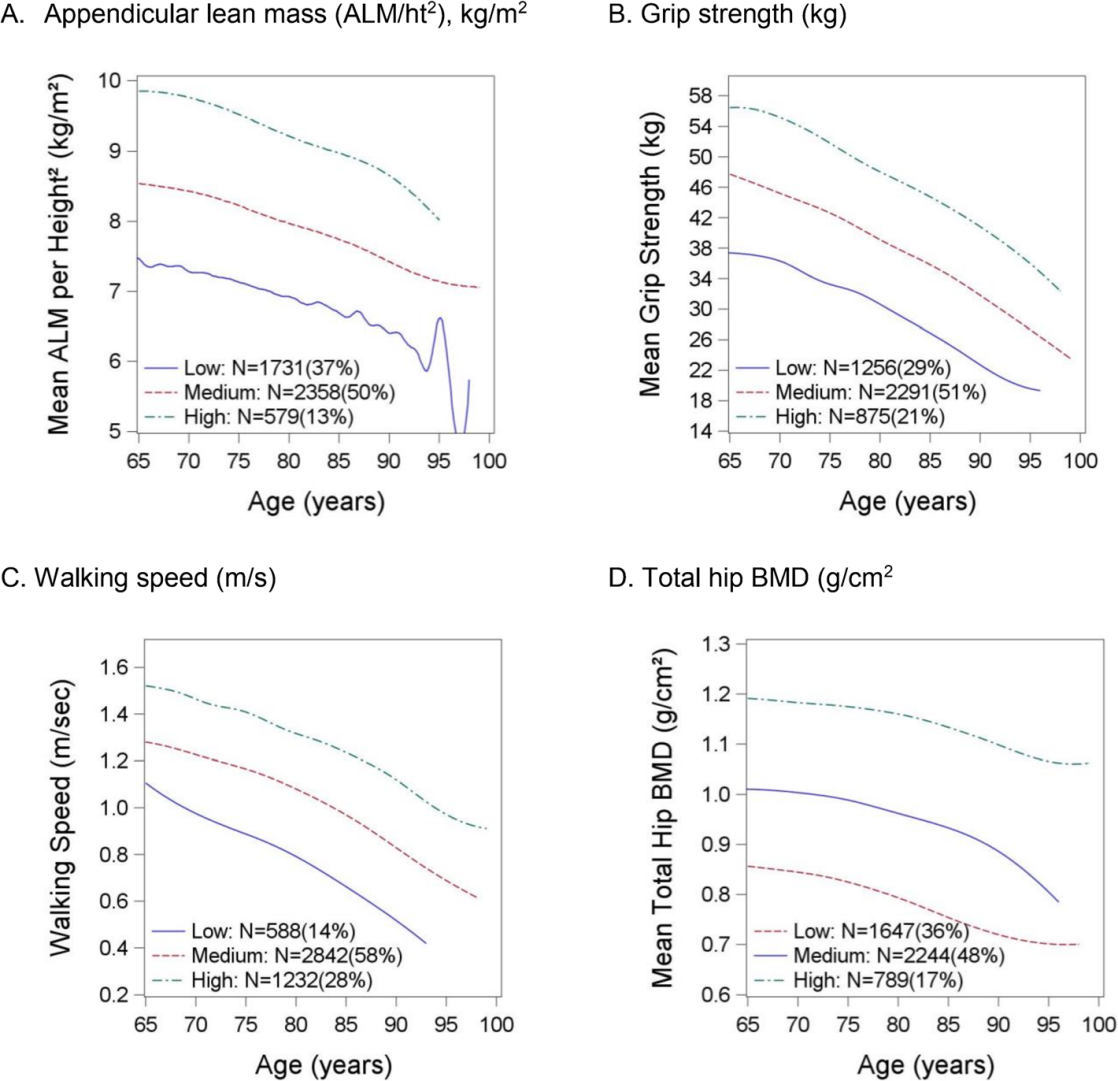
Individual trajectories in grip strength, walking speed, total hip BMD and ALM/ht2 in older men

Men in the MrOS cohort, and the analytic sample for this report, were relatively healthy with a mean age of 72.7 years (Table 1). The individual factor trajectories (for ALM/ht^2^, grip strength, walking speed and total hip BMD separately) are presented in Fig. 1. In general, the patterns identified are roughly similar for each factor: there appears to be decline in each trajectory with age, and there is no-cross over or convergence in the rate of change with age. Based on this descriptive information, we describe each factor to have three trajectories: “low”, “intermediate”, and “high”.

**Table 1:** Baseline characteristics (mean ± SD or N(%)) by inclusion in the analysis subset

	Analytic sample *N* = 4681	Not included in analytic sample *N* = 1313	*p*-value
Age (years)	72.7 ± 5.3	77 ± 6.5	< 0.0001
Walking speed (m/s)	1.23 ± 0.2	1.09 ± 0.25	< 0.0001
Grip strength (kg)	42.7 ± 8.2	37.6 ± 8.4	< 0.0001
PASE	151.7 ± 67.3	127.8 ± 68.4	< 0.0001
Total Hip BMD (g/cm^2^)	0.963 ± 0.14	0.939 ± 0.149	< 0.0001
Excellent/good health	4140 (88.4)	995 (75.9)	< 0.0001
One or more functional limitation^a^	769 (16.4)	465 (35.4)	< 0.0001
BMI (kg/m^2^)	27.4 ± 3.7	27.2 ± 4.1	0.0474
One or more medical conditions^b^	2825 (60.4)	973 (74.1)	< 0.0001
ALM/ht^2^ (kg/m^2^)	8.03 ± 0.9	7.79 ± 1.02	< 0.0001

^a^Functional limitations include: walking 2–3 blocks, climbing stairs, shopping, cooking meals and heavy housework

^b^One or more of the following: diabetes, stroke, myocardial infarction, non-skin cancer, high blood pressure, congestive heart failure and Parkinson’s disease

*PASE*Physical Activity Scale for the Elderly, *BMD* bone mineral density, *BMI* body mass index, *ALM* appendicular lean mass

**Fig. 2:**
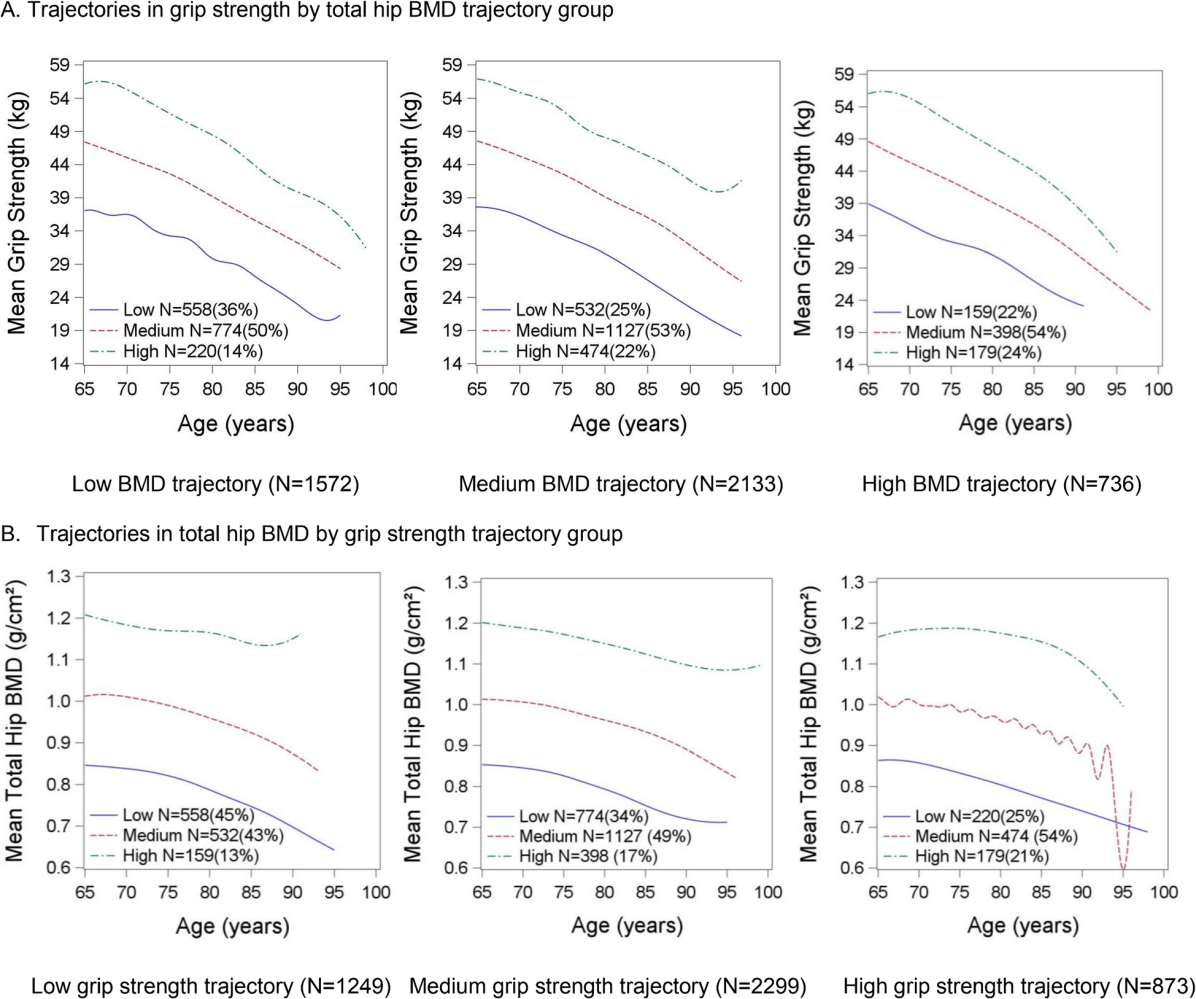
Joint trajectories in total hip BMD and grip strength in older men

As an illustrative example, the joint trajectories for total hip BMD and grip strength are shown in Fig. 2. In panel A, the trajectories for grip strength are shown for participants within the low BMD trajectory, the medium BMD trajectory and the high BMD trajectory. Some of the groups identified were rather small: only 159 men (3.6% of the total men in the model) were in the high BMD/low grip strength trajectory and only 179 men (4.0% of the total men in the model) were in the high BMD/high grip strength group; the most common placement was in the medium BMD/medium grip strength trajectory (*N* = 1127, 25.4% of the total men in the model). Figure 2, Panel B shows the trajectories in BMD across the low, medium and high grip strength groups. Differences in participant characteristics by the joint grip strength/BMD trajectory group are reported in Table 2. As expected, there are large differences in grip strength and BMD by the joint grip strength/BMD trajectory groups. In addition, aside from age, all of the other factors reported in Table 2 differed by joint grip strength/BMD trajectory group. Generally, those in the high grip strength trajectory groups, regardless of the BMD trajectory group, appeared to have the most favorable characteristics (e.g. fastest walking speed, higher physical activity, fewest ADL limitations).

**Table 2:** Characteristics of participants by joint trajectories in total hip BMD^a^ and grip strength in older men

	Low grip strength trajectory			Medium grip strength trajectory			High grip strength trajectory			Unable to complete grip	***p***-value
	Low BMD trajectory	Medium BMD trajectory	High BMD trajectory	Low BMD trajectory	Medium BMD trajectory	High BMD trajectory	Low BMD trajectory	Medium BMD trajectory	High BMD trajectory		
	*N* = 558	*N* = 532	*N* = 159	*N* = 774	N = 1127	*N* = 398	*N* = 220	*N* = 474	*N* = 179	*N* = 259	
Age (years)	72.3 ± 5.1	72.8 ± 5.3	72.6 ± 4.8	72.7 ± 5.3	73 ± 5.3	72.5 ± 5.5	72.8 ± 5.7	72.6 ± 5.4	72.4 ± 5.3	73.4 ± 5.6	0.143
Walking speed (m/s)	1.19 ± 0.22	1.20 ± 0.21	1.18 ± 0.22	1.24 ± 0.21	1.23 ± 0.21	1.22 ± 0.20	1.27 ± 0.22	1.29 ± 0.20	1.30 ± 0.21	1.20 ± 0.36	< 0001
Grip strength (kg)	34.6 ± 5	34.6 ± 5.5	35.1 ± 5.7	43.4 ± 5	43.5 ± 5.1	43.7 ± 5.1	52.7 ± 6.1	52.9 ± 5.3	52.8 ± 5.9	N/A	<.001
PASE	140.5 ± 64.7	147.4 ± 68.7	145.1 ± 69.3	150.6 ± 68.2	152.2 ± 65.7	155.1 ± 67.9	162.8 ± 62.5	159.1 ± 68.0	166.9 ± 70.8	151.3 ± 68.8	<.001
Total Hip BMD (g/cm^2^)	0.826 ± 0.08	0.991 ± 0.06	1.175 ± 0.08	0.826 ± 0.07	0.989 ± 0.06	1.173 ± 0.09	0.834 ± 0.07	0.988 ± 0.06	1.18 ± 0.08	0.955 ± 0.15	<.001
Excellent/good health	470 (84.2)	450 (84.6)	140 (88.1)	695 (89.8)	1003 (89.0)	351 (88.2)	203 (92.3)	448 (94.5)	171 (95.5)	208 (80.3)	<.001
One or more ADL limitation^a^	120 (21.5)	113 (21.2)	35 (22)	106 (13.7)	167 (14.8)	63 (15.8)	23 (10.5)	52 (11)	22 (12.3)	67 (25.9)	<.001
BMI (kg/m^2^)	26.2 ± 3.8	27.9 ± 3.7	29.9 ± 3.8	26 ± 3.1	27.8 ± 3.7	29 ± 4.1	26.1 ± 3	27.8 ± 3.4	29.2 ± 3.5	27.4 ± 3.7	<.001
One or more medical conditions**	370 (66.3)	361 (67.9)	108 (67.9)	431 (55.7)	690 (61.2)	242 (60.8)	105 (47.7)	252 (53.2)	106 (59.2)	160 (61.8)	<.001
ALM/ht2 (kg/m^2^)	7.6 ± 0.9	7.9 ± 0.9	8.3 ± 1	7.8 ± 0.7	8.1 ± 0.9	8.4 ± 0.9	8.1 ± 0.8	8.4 ± 0.9	8.7 ± 0.9	8.0 ± 1.0	<.001
Probability of identified trajectory	0.89 ± 0.2	0.85 ± 0.2	0.85 ± 0.2	0.85 ± 0.2	0.84 ± 0.2	0.85 ± 0.2	0.86 ± 0.2	0.87 ± 0.2	0.89 ± 0.2	N/A	N/A
Identified Trajectory probability < =0.5	14 (2.5)	20 (3.8)	3 (1.9)	23 (3)	41 (3.6)	15 (3.8)	3 (1.4)	15 (3.2)	4 (2.2)	N/A	0.59

^a^ 1 participant excluded from analysis due to missing BMD values at all follow up visits

Additional files 2, 3, 4, 5 and 6 show the joint trajectories for the other pair-wise trajectories examined, and characteristics of participants are reported for each pairwise trajectory group in Additional file 7. Overall, characteristics of participants varied by trajectory placement, although this appeared somewhat less pronounced with some non-significant differences for the ALM/ht^2^ and BMD joint trajectories.

Mean posterior probabilities, i.e. the mean of the probability of being assigned to trajectory based on the participant’s given data points, were reasonably high, ranging from 0.84–0.89, suggesting that most participants’ actual trajectory fit the modeled trajectory reasonably well.

## Discussion

We characterized patterns of change in bone density, strength, walking speed and appendicular lean mass. In contrast to our initial hypothesis that these analyses would reveal discrete categories of men with rapid decline in one or a combination of these musculoskeletal phenotypes, our results did not demonstrate such subgroups. In fact, the three groups of change characterized by the analysis (“high”, “medium”, and” low”) appeared to have little crossover or convergence of change with age, even when considering joint trajectories of change in these factors. The trajectory groups were associated with significant differences in other non-trajectory participant characteristics, although this was less pronounced for the ALM/ht^2^ and BMD joint trajectories than for the other individual and joint trajectories. Several other studies have examined changes in lean mass and muscle strength that occurs with older age in men [19, 34, 35] while some other reports have evaluated change in performance, lean mass, and bone density. However, few studies have sought to systematically identify concurrent patterns in change in these factors over time. We also found that men in low trajectories in one area tended to be in the low trajectories in another area (e.g. those in the low BMD trajectory tended to be in the low grip strength trajectory). Examining characteristic of men by trajectory group, men in low trajectories tended to have worse health status and more co-morbidities than those in the higher trajectories. It is possible that placement in the lower trajectories reflects less successful aging.

Unlike other analyses that have used a similar group-based trajectory modeling approach [36], we did not identify groups in which change appeared to cross-over or converge with age; nor were subsets of rapid change evident. Across the factors, the patterns of change identified were generally similar, with three groups declining over time at apparently comparable rates, although we did not formally test all pairwise difference between groups. This may be due to the fact that the components of the trajectories were only measured at three time points, which would limit our ability to see such non-linear change, such as rapid decline that might occur near the end of life or as a result of an acute illness or hospitalization. In addition, many other trajectory analyses are anchored to a specific event, such as change in disability patterns preceding death. In contrast, our data simply describes the decline in these factors with general aging rather than in response to acute events. Participants in MrOS were generally healthy at baseline and because the assessments used in the trajectory modeling were completed in men who returned to the clinic. Our data may also represent the healthiest subset of participants and underestimate trajectories that would be seen in more frail or sick men. The results of this study were somewhat surprising.

Our study used a data driven approach to find patterns of change in a large well characterized cohort of older men. This study provides a key descriptive background of possible categories of bone-muscle phenotypes. However, a number of limitations must be noted. First, the trajectory models are descriptive in nature and do not specifically test for different rates of change (e.g. slopes) or initial or ending values when determining group membership. This limits our ability to evaluate differences between trajectories unless we specifically test those differences post-hoc. Second, the posterior probabilities for trajectory placement were reasonably high, although some were closer to .8 suggesting that other models may more accurately reflect underlying patterns in the data, especially for very small subgroups. However, some joint trajectory groups had a non-trivial number of men with a relatively low probability of group membership (< 50%), suggesting that these method did not identify distinct trajectories that were followed by a small minority of men. Third, we have not tested whether these trajectories predict adverse health outcomes, nor whether any such associations are independent of the Year 7 value (that is, whether change per se versus where the participant ends up is important in predicting adverse outcomes). Such analyses will be completed in subsequent reports. Fourth, only an approximation of muscle mass [37, 38], ALM/ht^2^, was available at these MrOS visits. Other approaches that directly measure muscle mass, such as D_3_-creatine dilution [39, 40], may result in different findings. This is likely, as results from analyses at subsequent MrOS visits found that muscle mass assessed D_3_-creatine dilution was strongly related to physical performance and mobility outcomes, while DXA ALM/ht^2^ was not [39]. Finally, our graphical representations of the trajectories occasionally demonstrated highly variable patterns of change (for example, Additional file 5). This is due to small numbers of participants in some ages in trajectory groups. Larger numbers of participants would likely makes these trajectories less variable.

## Conclusion

Our description of trajectories of change with age in bone mineral density, grip strength, walking speed and appendicular lean mass found that groups identified by these methods appeared to have little crossover or convergence of change with age, even when considering joint trajectories of change in these factors. Subsequent studies will evaluate whether trajectories of change predict subsequent health outcomes including falls, fractures and mortality.

## Supplementary information

**Additional file 1: Figure S1.** Inclusion of participants in analysis.

**Additional file 2: Figure S2.** Joint trajectories in total hip BMD and walking speed in older men.

**Additional file 3: Figure S3.** Joint trajectories in ALM/ht^2^ and total hip BMD in older men.

**Additional file 4: Figure S4.** Joint trajectories in grip strength and walking speed in older men.

**Additional file 5: Figure S5.** Joint trajectories in grip strength and ALM/ht^2^ in older men.

**Additional file 6: Figure S6.** Joint trajectories in walking speed and ALM/ht^2^ in older men.

**Additional file 7: Table S1.** Characteristics of participants by joint trajectories in total hip BMD and walking speed in older men. **Table S2.** Characteristics of participants by joint trajectories in ALM/ht^2^ and total hip BMD in older men. **Table S3.** Characteristics of participants by joint trajectories in grip strength and walking speed in older men. **Table S4.** Characteristics of participants by joint trajectories in grip strength and ALM/ht^2^ in older men. **Table S5.** Characteristics of participants by joint trajectories in walking speed and ALM/ht^2^ in older men.

## Data Availability

The MrOS data are available here: https://mrosdata.sfcc-cpmc.net/ after agreeing to terms of data use. Files for this particular dataset are not available because a key variable, age, is an identifier (age over 89 is considered identifiable.) Access to the study data is available online after investigators agree to abide by terms of the data use agreement (as specified on the website above).

## Abbreviations

SAS: Statistical Analysis Software
MrOS: Osteoporotic Fractures in Men Study
BMD: Bone mineral density
DXA: Dual energy x-ray absorptiometry
Kg: kilograms
m/s: meters per second
g/cm^2^: grams per centimeter cubed
ALM: Appendicular lean mass
USA: United States of America
IL: Illinois
BMI: Body mass index
PASE: Physical Activity Scale for the Elderly
GBTM: Group based trajectory modeling
ANOVA: Analysis of Variance

## References

[1] Cooper R, Kuh D, Hardy R (2010). Objectively measured physical capability levels and mortality: systematic review and meta-analysis. Bmj.

[2] Cawthon PM, Ewing SK, McCulloch CE (2009). Loss of hip BMD in older men: the osteoporotic fractures in men (MrOS) study. J Bone Miner Res.

[3] Guralnik JM, Seeman TE, Tinetti ME, Nevitt MC, Berkman LF (1994). Validation and use of performance measures of functioning in a non-disabled older population: MacArthur studies of successful aging. Aging (Milan, Italy).

[4] Visser M, Goodpaster BH, Kritchevsky SB (2005). Muscle mass, muscle strength, and muscle fat infiltration as predictors of incident mobility limitations in well-functioning older persons. J Gerontol A Biol Sci Med Sci.

[5] Gill TM, Murphy TE, Barry LC, Allore HG (2009). Risk factors for disability subtypes in older persons. J Am Geriatr Soc.

[6] Tas U, Verhagen AP, Bierma-Zeinstra SM (2007). Incidence and risk factors of disability in the elderly: the Rotterdam study. Prev Med.

[7] Visser M, Deeg DJ, Lips P, Harris TB, Bouter LM (2000). Skeletal muscle mass and muscle strength in relation to lower-extremity performance in older men and women. J Am Geriatr Soc.

[8] Chan BK, Marshall LM, Winters KM, Faulkner KA, Schwartz AV, Orwoll ES (2007). Incident fall risk and physical activity and physical performance among older men: the osteoporotic fractures in men study. Am J Epidemiol.

[9] Cawthon PM, Fullman RL, Marshall L (2008). Physical performance and risk of hip fractures in older men. J Bone Miner Res.

[10] Leibson CL, Tosteson AN, Gabriel SE, Ransom JE, Melton LJ (2002). Mortality, disability, and nursing home use for persons with and without hip fracture: a population-based study. J Am Geriatr Soc.

[11] Magaziner J, Hawkes W, Hebel JR (2000). Recovery from hip fracture in eight areas of function. J Gerontol A Biol Sci Med Sci.

[12] Marks R, Allegrante JP, Ronald MacKenzie C, Lane JM (2003). Hip fractures among the elderly: causes, consequences and control. Ageing Res Rev.

[13] Lang T, Streeper T, Cawthon P, Baldwin K, Taaffe DR, Harris TB (2010). Sarcopenia: etiology, clinical consequences, intervention, and assessment. Osteoporos Int.

[14] Cawthon PM, Ewing SK, Mackey DC (2012). Change in hip bone mineral density and risk of subsequent fractures in older men. J Bone Miner Res.

[15] Cawthon PM, Patel S, Ewing SK (2017). Bone loss at the hip and subsequent mortality in older men: the osteoporotic fractures in men (MrOS) study. JBMR plus.

[16] Barbour KE, Lui LY, McCulloch CE (2016). Trajectories of lower extremity physical performance: effects on fractures and mortality in older women. J Gerontol A Biol Sci Med Sci.

[17] White DK, Neogi T, Nevitt MC (2013). Trajectories of gait speed predict mortality in well-functioning older adults: the health, aging and body composition study. J Gerontol A Biol Sci Med Sci.

[18] Barbour KE, Lui LY, McCulloch CE, Ensrud KE, Cawthon PM, Yaffe K, Barnes DE, Fredman L, Newman AB, Cummings SR, Cauley JA. Study of Osteoporotic Fractures. Trajectories of Lower Extremity Physical Performance: Effects on Fractures and Mortality in Older Women. J Gerontol A Biol Sci Med Sci. 2016;71(12):1609-15. Epub 2016 Apr 15. PubMed PMID: 27084313; PubMed Central PMCID: PMC5106858. 10.1093/gerona/glw071 PMC5106858 27084313

[19] Goodpaster BH, Park SW, Harris TB (2006). The loss of skeletal muscle strength, mass, and quality in older adults: the health, aging and body composition study. J Gerontol A Biol Sci Med Sci.

[20] Karasik D, Kiel DP (2010). Evidence for pleiotropic factors in genetics of the musculoskeletal system. Bone..

[21] Cruz-Jentoft AJ, Bahat G, Bauer J, Boirie Y, Bruyère O, Cederholm T, Cooper C, Landi F, Rolland Y, Sayer AA, Schneider SM, Sieber CC, Topinkova E, Vandewoude M, Visser M, Zamboni M. Writing Group for the European Working Group on Sarcopenia in Older People 2 (EWGSOP2), and the Extended Group for EWGSOP2. Sarcopenia: revised European consensus on definition and diagnosis. Age Ageing. 2019;48(1):16-31. 10.1093/ageing/afy169. Erratum in: Age Ageing. 2019;48(4):601. PubMed PMID: 30312372; PubMed Central PMCID: PMC6322506. 10.1093/ageing/afy169 PMC6322506 30312372

[22] Fielding RA, Vellas B, Evans WJ (2011). Sarcopenia: an undiagnosed condition in older adults. Current consensus definition: prevalence, etiology, and consequences. International working group on sarcopenia. J Am Med Dir Assoc.

[23] Studenski SA, Peters KW, Alley DE (2014). The FNIH sarcopenia project: rationale, study description, conference recommendations, and final estimates. J Gerontol A Biol Sci Med Sci.

[24] Studenski S, Perera S, Patel K (2011). Gait speed and survival in older adults. Jama..

[25] Rantanen T, Guralnik JM, Foley D (1999). Midlife hand grip strength as a predictor of old age disability. Jama.

[26] Baumgartner RN, Koehler KM, Gallagher D (1998). Epidemiology of sarcopenia among the elderly in New Mexico. Am J Epidemiol.

[27] Blank JB, Cawthon PM, Carrion-Petersen ML (2005). Overview of recruitment for the osteoporotic fractures in men study (MrOS). Contemp Clin Trials.

[28] Orwoll E, Blank JB, Barrett-Connor E (2005). Design and baseline characteristics of the osteoporotic fractures in men (MrOS) study--a large observational study of the determinants of fracture in older men. Contemp Clin Trials.

[29] Harkonen R, Harju R, Alaranta H (1993). Accuracy of the Jamar dynamometer. J Hand Ther.

[30] Washburn RA, Smith KW, Jette AM, Janney CA (1993). The physical activity scale for the elderly (PASE): development and evaluation. J ClinEpidemiol.

[31] Jones BL, Nagin DS, Roeder K (2001). A SAS Procedure Based on Mixture Models for Estimating Developmental Trajectories 1177/0049124101029003005. Sociol Method Res.

[32] Nagin DS, Odgers CL (2010). Group-based trajectory modeling in clinical research. Annu Rev Clin Psychol.

[33] Paul HCE, Marx BD (1996). Flexible smoothing with $B$-splines and penalties. Stat Sci.

[34] Kallman DA, Plato CC, Tobin JD (1990). The role of muscle loss in the age-related decline of grip strength: cross-sectional and longitudinal perspectives. J Gerontol.

[35] Rantanen T, Masaki K, Foley D, Izmirlian G, White L, Guralnik JM (1998). Grip strength changes over 27 yr in Japanese-American men. J Appl Physiol.

[36] Gill TM, Gahbauer EA, Han L, Allore HG (2010). Trajectories of disability in the last year of life. N Engl J Med.

[37] Clark BC, Tavoian D, Goodpaster BH, Cawthon PM, Hansen RD, Manini TM (2018). Comment on: "pitfalls in the measurement of muscle mass: a need for a reference standard" by Buckinx et al. J Cachexia Sarcopenia Muscle.

[38] Buckinx F, Landi F, Cesari M, Fielding RA, Visser M, Engelke K, Maggi S, Dennison E, Al-Daghri NM, Allepaerts S, Bauer J, Bautmans I, Brandi ML, Bruyère O, Cederholm T, Cerreta F, Cherubini A, Cooper C, Cruz-Jentoft A, McCloskey E, Dawson-Hughes B, Kaufman JM, Laslop A, Petermans J, Reginster JY, Rizzoli R, Robinson S, Rolland Y, Rueda R, Vellas B, Kanis JA. Pitfalls in the measurement of muscle mass: a need for a reference standard. J Cachexia Sarcopenia Muscle. 2018;9(2):269-78. 10.1002/jcsm.12268. Epub 2018 Jan 19. PubMed PMID: 29349935; PubMed Central PMCID: PMC5879987. 10.1002/jcsm.12268 PMC5879987 29349935

[39] Cawthon PM, Orwoll ES, Peters KE (2019). Strong relation between muscle mass determined by D3-creatine dilution, physical performance, and incidence of falls and mobility limitations in a prospective cohort of older men. J Gerontol A Biol Sci Med Sci.

[40] Duchowny KA, Peters KE, Cummings SR (2020). Association of change in muscle mass assessed by D3 -creatine dilution with changes in grip strength and walking speed. J Cachexia Sarcopenia Muscle.

