# Supplementary figures and images for: Individual and joint trajectories of change in bone, lean mass and physical performance in older men
Source: BMC Geriatr. 2020 May 5;20:161. doi: 10.1186/s12877-020-01560-5 (PMC7201689; doi:10.1186/s12877-020-01560-5)

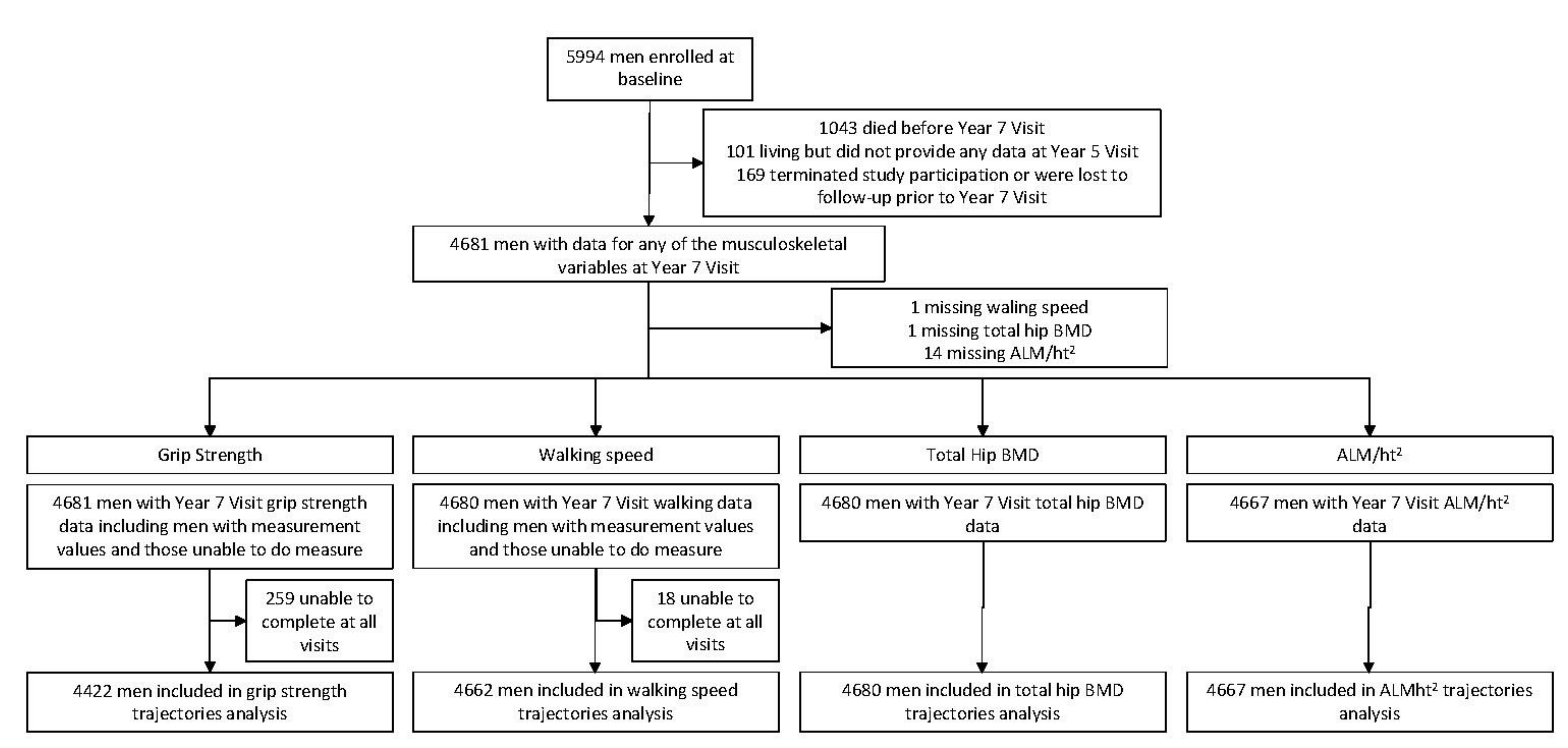

Supplement: Supplementary file 1 — Additional file 1: Figure S1. Inclusion of participants in analysis. [file 12877_2020_1560_MOESM1_ESM.png]

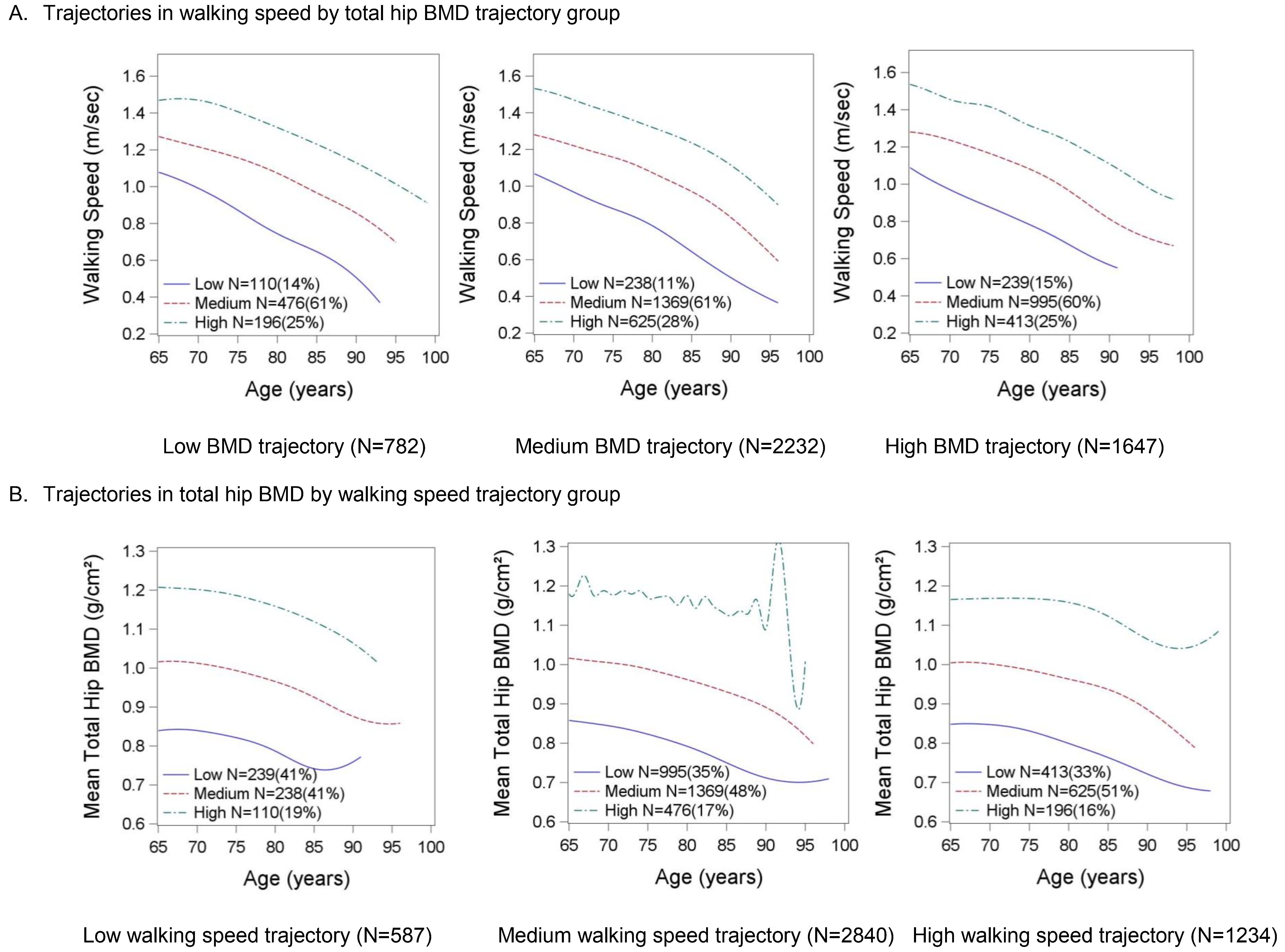

Supplement: Supplementary file 2 — Additional file 2: Figure S2. Joint trajectories in total hip BMD and walking speed in older men. [file 12877_2020_1560_MOESM2_ESM.png]

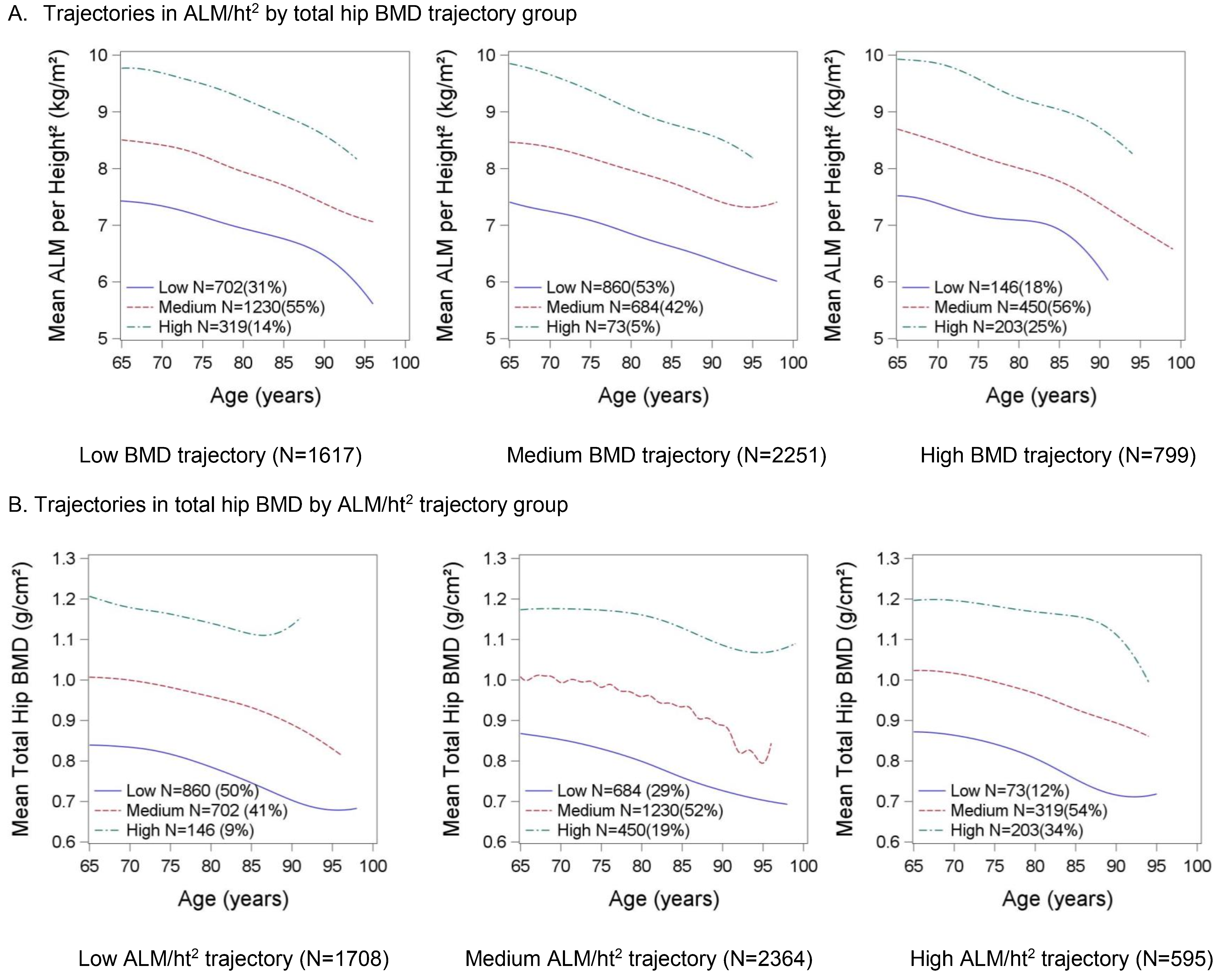

Supplement: Supplementary file 3 — Additional file 3: Figure S3. Joint trajectories in ALM/ht2 and total hip BMD in older men. [file 12877_2020_1560_MOESM3_ESM.png]

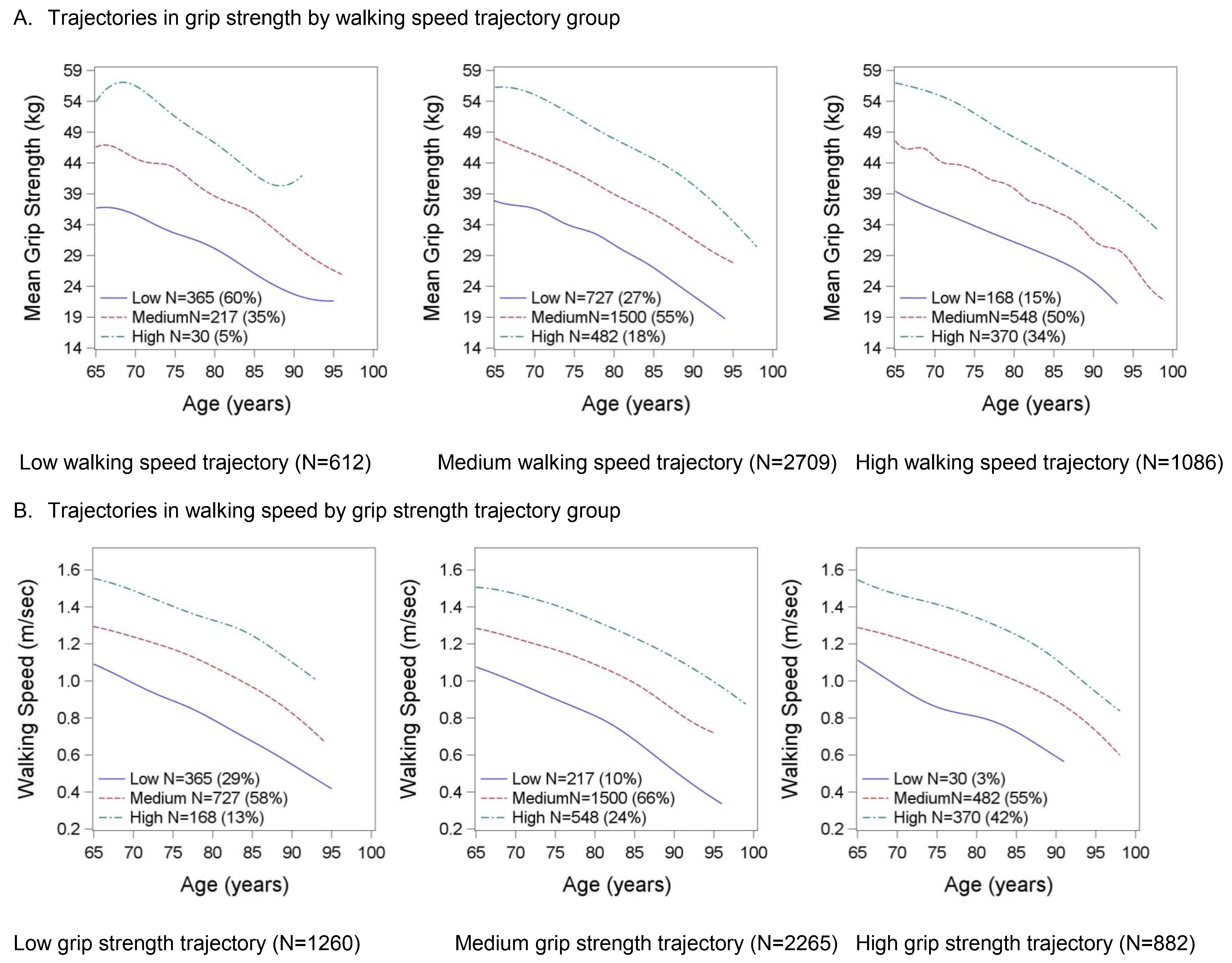

Supplement: Supplementary file 4 — Additional file 4: Figure S4. Joint trajectories in grip strength and walking speed in older men. [file 12877_2020_1560_MOESM4_ESM.png]

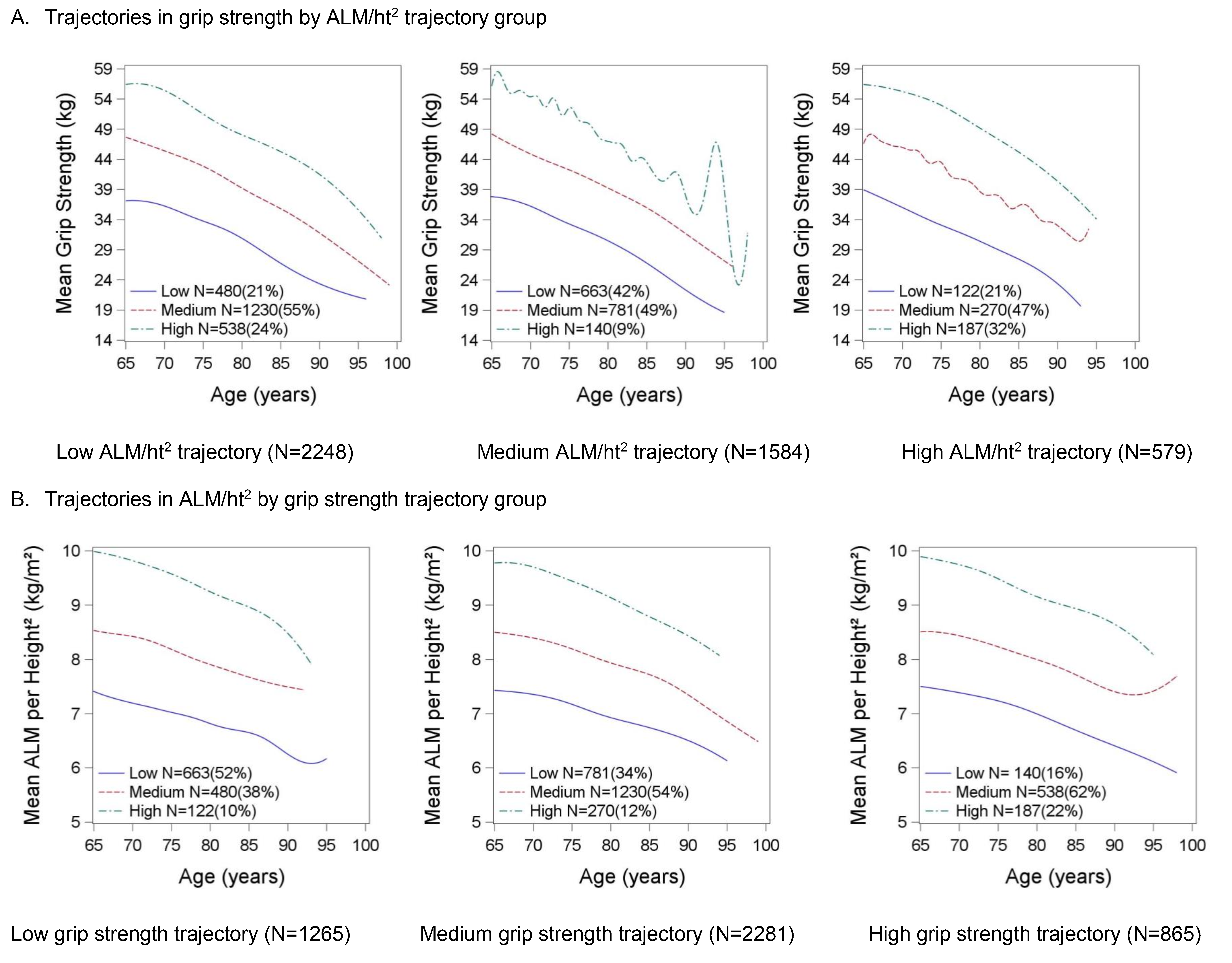

Supplement: Supplementary file 5 — Additional file 5: Figure S5. Joint trajectories in grip strength and ALM/ht2 in older men. [file 12877_2020_1560_MOESM5_ESM.png]

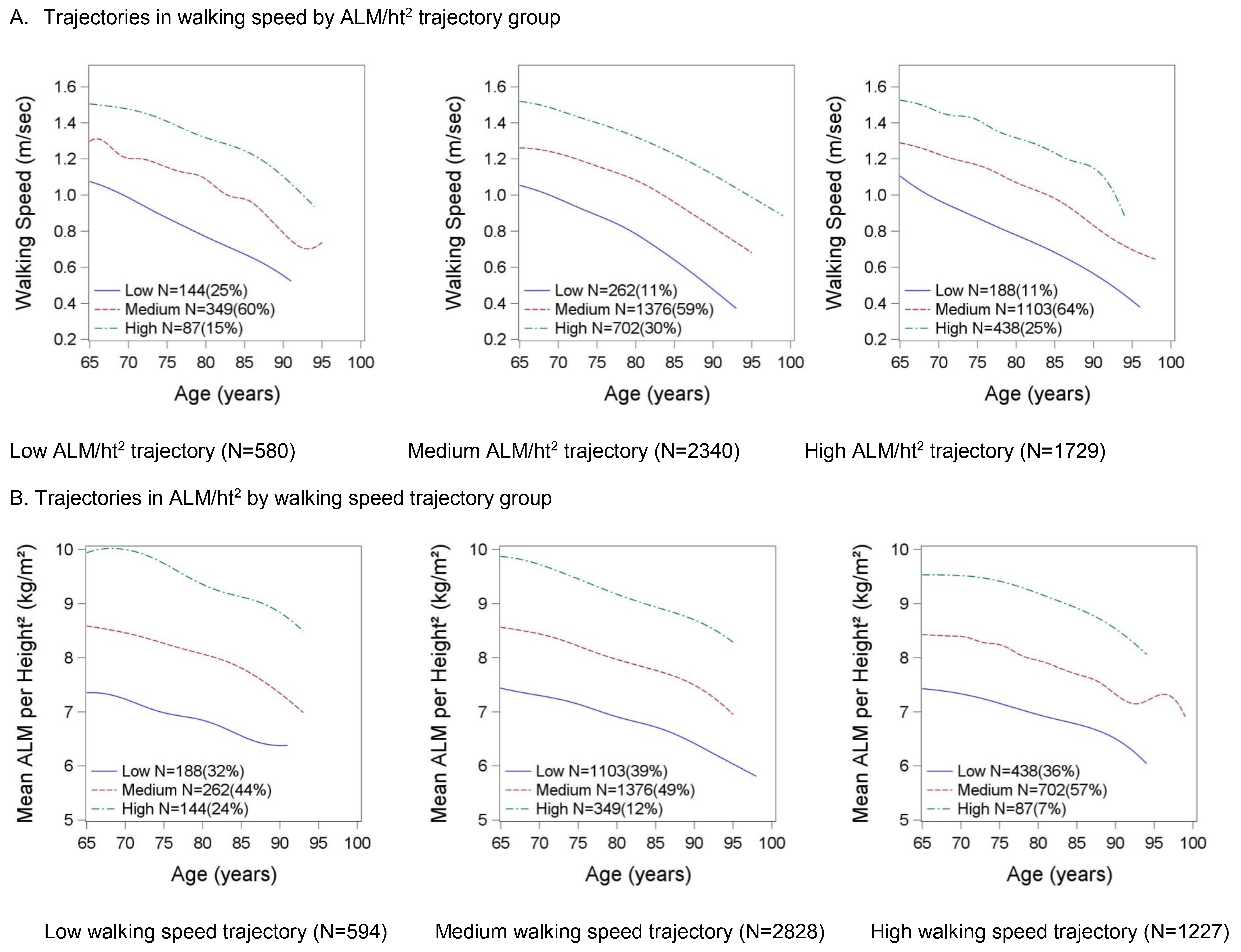

Supplement: Supplementary file 6 — Additional file 6: Figure S6. Joint trajectories in walking speed and ALM/ht2 in older men. [file 12877_2020_1560_MOESM6_ESM.png]
