# Supplementary material for: Individual and joint trajectories of change in bone, lean mass and physical performance in older men
Source: BMC Geriatr. 2020 May 5;20:161. doi: 10.1186/s12877-020-01560-5 (PMC7201689; doi:10.1186/s12877-020-01560-5)
Supplement: Supplementary file 7 — Additional file 7: Table S1. Characteristics of participants by joint trajectories in total hip BMD and walking speed in older men. Table S2. Characteristics of participants by joint trajectories in ALM/ht2 and total hip BMD in older men. Table S3. Characteristics of participants by joint trajectories in grip strength and walking speed in older men. Table S4. Characteristics of participants by joint trajectories in grip strength and ALM/ht2 in older men. Table S5. Characteristics of participants by joint trajectories in walking speed and ALM/ht2 in older men. [file 12877_2020_1560_MOESM7_ESM.docx]

|  | **Low walking speed trajectory** | | | **Medium walking speed trajectory** | | | **High walking speed trajectory** | | |  | **p-value** |
| --- | --- | --- | --- | --- | --- | --- | --- | --- | --- | --- | --- |
|  | **Low BMD trajectory** | **Medium BMD trajectory** | **High BMD trajectory** | **Low BMD trajectory** | **Medium BMD trajectory** | **High BMD trajectory** | **Low BMD trajectory** | **Medium BMD trajectory** | **High BMD trajectory** | **Unable to complete walking speed** |  |
|  | N=239 | N=238 | N=110 | N= 995 | N=1369 | N= 476 | N= 413 | N= 625 | N=196 | (N= 18) |  |
| Age (years) | 72.2 ± 4.9 | 72.2 ± 5.1 | 72.5 ± 5 | 72.4 ± 5.3 | 72.7 ± 5.2 | 72.2 ± 5.3 | 73.2 ± 5.5 | 73.4 ± 5.6 | 73.3 ± 5.7 | 75.4 ± 5.3 | <.001 |
| Walking speed (m/s) | 0.96 ± 0.17 | 0.96 ± 0.18 | 0.96 ± 0.17 | 1.21 ± 0.16 | 1.2 ± 0.15 | 1.2 ± 0.15 | 1.43 ± 0.17 | 1.42 ± 0.17 | 1.42 ± 0.17 | N/A | <.001 |
| Grip strength (kg) | 38.5 ± 8.3 | 41.1 ± 7.9 | 40.1 ± 8.4 | 41.8 ± 8.0 | 43 ± 8.0 | 44.3 ± 8.1 | 43 ± 7.9 | 44.4 ± 8.3 | 45.2 ± 7.8 | 37.1 ± 10 | <.001 |
| PASE | 128.1 ± 67.3 | 136.5 ± 67.4 | 137.1 ± 77.0 | 149 ± 66.1 | 151.8 ± 66.7 | 156.8 ± 66.7 | 159.7 ± 64.7 | 162.7 ± 67.2 | 160.6 ± 67.0 | 108.2 ± 62 | <.001 |
| Total Hip BMD (g/cm^2^) | 0.833 ± 0.07 | 1.004 ± 0.06 | 1.191 ± 0.09 | 0.827 ± 0.07 | 0.989 ± 0.06 | 1.176 ± 0.09 | 0.823 ± 0.07 | 0.983 ± 0.06 | 1.159 ± 0.07 | 1.015 ± 0.113 | <.001 |
| Excellent/good health | 163 (68.2) | 174 (73.1) | 80 (72.7) | 887 (89.1) | 1214 (88.7) | 429 (90.1) | 393 (95.2) | 597 (95.5) | 188 (95.9) | 13 (72.2) | <.001 |
| One or more ADL limitation* | 94 (39.3) | 101 (42.4) | 53 (48.2) | 132 (13.3) | 211 (15.4) | 69 (14.5) | 39 (9.4) | 51 (8.2) | 10 (5.1) | 7 (38.9) | <.001 |
| BMI (kg/m^2^) | 27.4 ± 4.2 | 30.2 ± 4.4 | 32.2 ± 4.4 | 26.1 ± 3.3 | 27.8 ± 3.5 | 29.3 ± 3.7 | 25.2 ± 2.7 | 26.9 ± 3.0 | 27.6 ± 3 | 28.5 ± 3.5 | <.001 |
| One or more medical conditions** | 174 (72.8) | 176 (73.9) | 87 (79.1) | 576 (57.9) | 860 (62.8) | 300 (63.0) | 208 (50.4) | 324 (51.8) | 105 (53.6) | 14 (77.8) | <.001 |
| ALM/ht^2^ (kg/m^2^) | 7.8 ± 1.0 | 8.5 ± 1.0 | 8.9 ± 1.1 | 7.8 ± 0.8 | 8.1 ± 0.9 | 8.4 ± 0.9 | 7.7 ± 0.7 | 8.0 ± 0.8 | 8.2 ± 0.7 | 7.9 ± 0.9 | <.001 |
| Probability of identified trajectory | 0.84 ± 0.17 | 0.82 ± 0.17 | 0.83 ± 0.18 | 0.83 ± 0.16 | 0.82 ± 0.17 | 0.85 ± 0.16 | 0.82 ± 0.18 | 0.83 ± 0.17 | 0.85 ± 0.17 | N/A | N/A |
| Identified Trajectory probability < =0.5 | 8 (3.3) | 9 (3.8) | 3 (2.7) | 35 (3.5) | 64 (4.7) | 19 (4) | 20 (4.8) | 20 (3.2) | 5 (2.6) | N/A | 0.67 |

Supplemental Table 1. Characteristics of participants by joint trajectories in total hip BMD and walking speed in older men

|  | **Low ALM/ht^2^ trajectory** | | | **Medium ALM/ht^2^ trajectory** | | | **High ALM/ht^2^ trajectory** | | | **p-value** |
| --- | --- | --- | --- | --- | --- | --- | --- | --- | --- | --- |
|  | **Low BMD trajectory** | **Medium BMD trajectory** | **High BMD trajectory** | **Low BMD trajectory** | **Medium BMD trajectory** | **High BMD trajectory** | **Low BMD trajectory** | **Medium BMD trajectory** | **High BMD trajectory** |  |
|  | N=860 | N=702 | N=146 | N=684 | N=1230 | N=450 | N=73 | N=319 | N= 203 |  |
| Age (years) | 72.5 ± 5.3 | 73.2 ± 5.3 | 71.9 ± 5.2 | 72.7 ± 5.2 | 72.8 ± 5.4 | 72.9 ± 5.4 | 72.5 ± 5.7 | 72.4 ± 5.2 | 72.2 ± 5.4 | 0.034 |
| Walking speed (m/s) | 1.23 ± 0.22 | 1.24 ± 0.21 | 1.25 ± 0.22 | 1.23 ± 0.21 | 1.24 ± 0.21 | 1.24 ± 0.21 | 1.19 ± 0.22 | 1.20 ± 0.22 | 1.18 ± 0.21 | 0.003 |
| Grip strength (kg) | 40.3 ± 7.8 | 40.8 ± 7.4 | 42.7 ± 6.9 | 42.9 ± 8.1 | 43.9 ± 8.3 | 43.9 ± 8.3 | 43.2 ± 8.5 | 45.6 ± 8.4 | 44.9 ± 8.6 | <0.001 |
| PASE | 148.9 ± 67.8 | 149.8 ± 67.8 | 155.2 ± 61.8 | 149.4 ± 65.0 | 155.5 ± 66.8 | 155.4 ± 68.9 | 142.1 ± 68.1 | 150.9 ± 67.4 | 153.7 ± 72.4 | 0.210 |
| Total Hip BMD (g/cm^2^) | 0.818 ± 0.08 | 0.984 ± 0.06 | 1.165 ± 0.08 | 0.834 ± 0.07 | 0.987 ± 0.06 | 1.167 ± 0.07 | 0.842 ± 0.06 | 0.997 ± 0.07 | 1.189 ± 0.1 | <0.001 |
| Excellent/good health | 755 (87.8) | 617 (87.9) | 135 (92.5) | 607 (88.7) | 1105 (89.8) | 403 (89.6) | 61 (83.6) | 277 (86.8) | 173 (85.2) | 0.237 |
| One or more ADL limitation* | 142 (16.5) | 103 (14.7) | 21 (14.4) | 98 (14.3) | 198 (16.1) | 67 (14.9) | 16 (21.9) | 66 (20.7) | 47 (23.2) | 0.024 |
| BMI (kg/m^2^) | 24.4 ± 2.5 | 25.4 ± 2.5 | 26.3 ± 2.6 | 27.4 ± 2.8 | 28.1 ± 2.8 | 28.6 ± 2.9 | 31.2 ± 3.3 | 32.1 ± 3.9 | 33.1 ± 3.8 | <0.001 |
| One or more medical conditions** | 505 (58.7) | 425 (60.5) | 80 (54.8) | 386 (56.4) | 733 (59.6) | 276 (61.3) | 47 (64.4) | 212 (66.5) | 151 (74.4) | <0.001 |
| ALM/ht^2^ (kg/m^2^) | 7.1 ± 0.5 | 7.2 ± 0.4 | 7.3 ± 0.4 | 8.2 ± 0.5 | 8.2 ± 0.5 | 8.3 ± 0.5 | 9.4 ± 0.7 | 9.5 ± 0.7 | 9.6 ± 0.7 | <0.001 |
| Probability of identified trajectory | 0.92 ± 0.14 | 0.86 ± 0.17 | 0.86 ± 0.17 | 0.88 ± 0.15 | 0.87 ± 0.16 | 0.89 ± 0.15 | 0.82 ± 0.19 | 0.88 ± 0.16 | 0.91 ± 0.15 | N/A |
| Identified Trajectory probability <=0.5 | 6 (0.7) | 20 (2.8) | 5 (3.4) | 16 (2.3) | 32 (2.6) | 12 (2.7) | 2 (2.7) | 9 (2.8) | 3 (1.5) | 0.09 |

Supplemental Table 2. Characteristics of participants by joint trajectories in ALM/ht^2^ and total hip BMD in older men

Supplemental Table 3. Characteristics of participants by joint trajectories in grip strength and walking speed in older men

|  | **Low grip strength trajectory** | | | **Medium grip strength trajectory** | | | **High grip strength trajectory** | | | **Unable to complete grip or walking speed** | **p-value** |
| --- | --- | --- | --- | --- | --- | --- | --- | --- | --- | --- | --- |
|  | **Low walking speed trajectory** | **Medium walking speed trajectory** | **High walking speed trajectory** | **Low walking speed trajectory** | **Medium walking speed trajectory** | **High walking speed trajectory** | **Low walking speed trajectory** | **Medium walking speed trajectory** | **High walking speed trajectory** |  |  |
|  | N=365 | N=727 | N=168 | N=217 | N=1500 | N=548 | N=30 | N=482 | N=370 | N= 273 |  |
| Age (years) | 72.3 ± 4.9 | 72.5 ± 5.2 | 72.8 ± 5.3 | 72.2 ± 5.1 | 72.6 ± 5.3 | 73.4 ± 5.7 | 71.3 ± 6.2 | 72.2 ± 5.2 | 73.3 ± 5.7 | 73.5 ± 5.6 | <.001 |
| Walking speed (m/s) | 0.98 ± 0.17 | 1.22 ± 0.15 | 1.46 ± 0.16 | 0.97 ± 0.17 | 1.20 ± 0.15 | 1.42 ± 0.17 | 1.01 ± 0.16 | 1.21 ± 0.16 | 1.43 ± 0.17 | N/A | <.001 |
| Grip strength (kg) | 34.8 ± 5.8 | 34.7 ± 5.1 | 35.1 ± 4.9 | 44.3 ± 5 | 43.7 ± 5.1 | 42.7 ± 5.2 | 54.2 ± 6 | 52.9 ± 5.4 | 52.3 ± 6 | N/A | <.001 |
| PASE | 128.6 ± 68.4 | 150.4 ± 64.9 | 152.5 ± 67.7 | 142.6 ± 69.8 | 151.0 ± 66.5 | 160.3 ± 65.8 | 158.2 ± 71.4 | 156.5 ± 67.4 | 168.0 ± 66.6 | 149.1 ± 69.2 | <.001 |
| Total Hip BMD (g/cm^2^) | 0.953 ± 0.15 | 0.942 ± 0.14 | 0.938 ± 0.14 | 0.982 ± 0.14 | 0.968 ± 0.14 | 0.952 ± 0.13 | 0.983 ± 0.15 | 0.989 ± 0.13 | 0.979 ± 0.13 | 0.959 ± 0.14 | <.001 |
| Excellent/good health | 268 (73.4) | 641 (88.2) | 157 (93.5) | 156 (71.9) | 1344 (89.6) | 523 (95.4) | 25 (83.3) | 447 (92.7) | 360 (97.3) | 218 (79.9) | <.001 |
| One or more ADL limitation* | 140 (38.4) | 119 (16.4) | 17 (10.1) | 78 (35.9) | 208 (13.9) | 38 (6.9) | 16 (53.3) | 52 (10.8) | 27 (7.3) | 73 (26.7) | <.001 |
| BMI (kg/m^2^) | 28.8 ± 4.7 | 27.2 ± 3.6 | 25.8 ± 2.9 | 29.7 ± 4.8 | 27.4 ± 3.6 | 26.3 ± 3.1 | 30.5 ± 3.5 | 27.9 ± 3.6 | 26.8 ± 3 | 27.5 ± 3.7 | <.001 |
| One or more medical conditions** | 276 (75.6) | 476 (65.5) | 96 (57.1) | 152 (70.0) | 899 (59.9) | 292 (53.3) | 22 (73.3) | 270 (56.0) | 171 (46.2) | 170 (62.3) | <.001 |
| ALM/ht^2^ (kg/m^2^) | 8.1 ± 1.1 | 7.7 ± 0.9 | 7.6 ± 0.7 | 8.4 ± 1 | 8.1 ± 0.9 | 7.8 ± 0.7 | 8.8 ± 1.1 | 8.4 ± 0.9 | 8.2 ± 0.8 | 8.0 ± 1 | <.001 |
| Probability of identified trajectory | 0.83 ± 0.18 | 0.81 ± 0.18 | 0.75 ± 0.19 | 0.77 ± 0.19 | 0.8 ± 0.17 | 0.79 ± 0.19 | 0.77 ± 0.2 | 0.8 ± 0.17 | 0.83 ± 0.18 | N/A | N/A |
| Identified Trajectory probability < =0.5 | 17 (4.7) | 51 (7) | 17 (10.1) | 19 (8.8) | 89 (5.9) | 48 (8.8) | 3 (10) | 29 (6) | 21 (5.7) | N/A | 0.09 |

Supplemental Table 4. Characteristics of participants by joint trajectories in grip strength and ALM/ht^2^ in older men

|  | **Low grip strength trajectory** | | | **Medium grip strength trajectory** | | | **High grip strength trajectory** | | | **Unable to complete grip strength** | **p-value** |
| --- | --- | --- | --- | --- | --- | --- | --- | --- | --- | --- | --- |
|  | **Low ALM/ht^2^ trajectory** | **Medium ALM/ht^2^ trajectory** | **High ALM/ht^2^ trajectory** | **Low ALM/ht^2^ trajectory** | **Medium ALM/ht^2^ trajectory** | **High ALM/ht^2^ trajectory** | **Low ALM/ht^2^ trajectory** | **Medium ALM/ht^2^ trajectory** | **High ALM/ht^2^ trajectory** |  |  |
|  | N=663 | N=480 | N=122 | N=781 | N=1230 | N=270 | N=140 | N= 538 | N= 187 | N=257 |  |
| Age (years) | 72.4 ± 5.1 | 72.5 ± 5.1 | 72.3 ± 5.0 | 72.7 ± 5.3 | 73.0 ± 5.4 | 72.4 ± 5.3 | 72.8 ± 5.7 | 72.6 ± 5.3 | 72.2 ± 5.6 | 73.4 ± 5.7 | 0.130 |
| Walking speed (m/s) | 1.2 ± 0.22 | 1.19 ± 0.21 | 1.13 ± 0.22 | 1.25 ± 0.21 | 1.23 ± 0.2 | 1.18 ± 0.19 | 1.29 ± 0.20 | 1.30 ± 0.20 | 1.26 ± 0.20 | 1.23 ± 0.25 | <.001 |
| Grip strength (kg) | 34.8 ± 5.2 | 34.6 ± 5.5 | 35.1 ± 5.3 | 43.6 ± 4.9 | 43.4 ± 5.2 | 44.1 ± 5.1 | 51.9 ± 5.8 | 53 ± 5.6 | 53.2 ± 5.7 | N/A | <.001 |
| PASE | 143.7 ± 67 | 146.4 ± 65.9 | 143.3 ± 68.5 | 152.6 ± 67.3 | 152.4 ± 65.7 | 149.9 ± 71.7 | 159.5 ± 62.7 | 163.3 ± 68.2 | 160.4 ± 68.7 | 151.9 ± 68.4 | <.001 |
| Total Hip BMD (g/cm^2^) | 0.905 ± 0.13 | 0.969 ± 0.13 | 1.032 ± 0.15 | 0.929 ± 0.13 | 0.972 ± 0.13 | 1.042 ± 0.14 | 0.937 ± 0.12 | 0.987 ± 0.13 | 1.033 ± 0.13 | 0.955 ± 0.14 | <.001 |
| Excellent/good health | 566 (85.4) | 413 (86.0) | 97 (79.5) | 700 (89.6) | 1105 (89.8) | 231 (85.6) | 133 (95.0) | 502 (93.3) | 179 (95.7) | 208 (80.9) | <.001 |
| One or more ADL limitation* | 126 (19) | 107 (22.3) | 33 (27) | 104 (13.3) | 172 (14) | 56 (20.7) | 12 (8.6) | 61 (11.3) | 23 (12.3) | 65 (25.3) | <.001 |
| BMI (kg/m^2^) | 25.2 ± 2.8 | 28.6 ± 2.9 | 33.6 ± 4.0 | 24.9 ± 2.4 | 27.9 ± 2.9 | 32.3 ± 4.0 | 24.7 ± 2.5 | 27.3 ± 2.7 | 31.2 ± 3.4 | 27.3 ± 3.7 | <.001 |
| One or more medical conditions** | 436 (65.8) | 316 (65.8) | 95 (77.9) | 428 (54.8) | 736 (59.8) | 188 (69.6) | 71 (50.7) | 279 (51.9) | 108 (57.8) | 158 (61.5) | <.001 |
| ALM/ht^2^ (kg/m^2^) | 7.1 ± 0.5 | 8.3 ± 0.5 | 9.6 ± 0.8 | 7.2 ± 0.4 | 8.2 ± 0.5 | 9.5 ± 0.6 | 7.3 ± 0.4 | 8.3 ± 0.5 | 9.5 ± 0.7 | 8.0 ± 1 | <.001 |
| Probability of identified trajectory | 0.88 ± 0.15 | 0.84 ± 0.18 | 0.86 ± 0.17 | 0.84 ± 0.17 | 0.84 ± 0.17 | 0.81 ± 0.18 | 0.8 ± 0.19 | 0.87 ± 0.16 | 0.9 ± 0.14 | N/A | N/A |
| Identified Trajectory probability < =0.5 | 5 (0.8) | 26 (5.4) | 5 (4.1) | 31 (4) | 48 (3.9) | 17 (6.3) | 8 (5.7) | 18 (3.3) | 1 (0.5) | N/A | <.001 |

|  | **Low walking speed trajectory** | | | **Medium walking speed trajectory** | | | **High walking speed trajectory** | | | **Unable to complete walking speed** | **p-value** |  |
| --- | --- | --- | --- | --- | --- | --- | --- | --- | --- | --- | --- | --- |
|  | **Low ALM/ht^2^ trajectory** | **Medium ALM/ht^2^ trajectory** | **High ALM/ht^2^ trajectory** | **Low ALM/ht^2^ trajectory** | **Medium ALM/ht^2^ trajectory** | **High ALM/ht^2^ trajectory** | **Low ALM/ht^2^ trajectory** | **Medium ALM/ht^2^ trajectory** | **High ALM/ht^2^ trajectory** |  |  |  |
|  | N=188 | N=262 | N=144 | N=1103 | N=1376 | N=349 | N=438 | N=702 | N=87 | (N= 18) |  |  |
| Age (years) | 72.1 ± 5.1 | 72.3 ± 4.9 | 72 ± 4.8 | 72.8 ± 5.3 | 72.5 ± 5.2 | 72.1 ± 5.3 | 72.8 ± 5.4 | 73.7 ± 5.6 | 73.6 ± 6.1 | 75.4 ± 5.3 | <.001 |  |
| Walking speed (m/s) | 0.96 ± 0.19 | 0.97 ± 0.17 | 0.97 ± 0.17 | 1.2 ± 0.16 | 1.2 ± 0.15 | 1.21 ± 0.15 | 1.43 ± 0.17 | 1.42 ± 0.17 | 1.45 ± 0.15 | N/A | <.001 |  |
| Grip strength (kg) | 37.7 ± 8.1 | 40.5 ± 8.0 | 42.6 ± 8.5 | 40.8 ± 7.4 | 43.7 ± 8.2 | 45.3 ± 8.2 | 41.9 ± 7.2 | 44.8 ± 8.2 | 48.3 ± 8.4 | 37.1 ± 10.0 | <.001 |  |
| PASE | 131.9 ± 67.3 | 131.2 ± 65.9 | 144 ± 74.3 | 149.9 ± 67.1 | 153.8 ± 66.5 | 151.7 ± 67.5 | 156.8 ± 66.5 | 163.4 ± 64.4 | 161.6 ± 73 | 108.2 ± 62 | <.001 |  |
| Total Hip BMD (g/cm^2^) | 0.905 ± 0.13 | 0.972 ± 0.14 | 1.064 ± 0.14 | 0.925 ± 0.14 | 0.976 ± 0.13 | 1.031 ± 0.14 | 0.918 ± 0.13 | 0.974 ± 0.13 | 1.025 ± 0.12 | 1.015 ± 0.11 | <.001 |  |
| Excellent/good health | 136 (72.3) | 190 (72.5) | 101 (70.1) | 977 (88.6) | 1236 (89.8) | 308 (88.3) | 416 (95.0) | 671 (95.6) | 85 (97.7) | 13 (72.2) | <.001 |  |
| One or more ADL limitation* | 71 (37.8) | 108 (41.2) | 66 (45.8) | 160 (14.5) | 197 (14.3) | 56 (16) | 35 (8) | 53 (7.5) | 5 (5.7) | 7 (38.9) | <.001 |  |
| BMI (kg/m^2^) | 25.6 ± 2.9 | 29.7 ± 3.1 | 34.6 ± 4.2 | 25.1 ± 2.6 | 28.1 ± 2.8 | 32.0 ± 3.5 | 24.5 ± 2.4 | 27.1 ± 2.5 | 30.2 ± 2.9 | 28.5 ± 3.5 | <.001 |  |
| One or more medical conditions** | 142 (75.5) | 184 (70.2) | 117 (81.3) | 657 (59.6) | 827 (60.1) | 238 (68.2) | 216 (49.3) | 371 (52.8) | 48 (55.2) | 14 (77.8) | <.001 |  |
| ALM/ht^2^ (kg/m^2^) | 7.2 ± 0.5 | 8.4 ± 0.5 | 9.8 ± 0.9 | 7.2 ± 0.5 | 8.3 ± 0.5 | 9.5 ± 0.6 | 7.2 ± 0.4 | 8.2 ± 0.5 | 9.3 ± 0.5 | 7.9 ± 0.9 | <.001 |  |
| Probability of identified trajectory | 0.81 ± 0.19 | 0.81 ± 0.18 | 0.83 ± 0.17 | 0.83 ± 0.16 | 0.81 ± 0.17 | 0.82 ± 0.17 | 0.81 ± 0.18 | 0.83 ± 0.17 | 0.81 ± 0.18 | N/A | N/A |  |
| Identified Trajectory probability < =0.5 | 11 (5.9) | 12 (4.6) | 5 (3.5) | 42 (3.8) | 90 (6.5) | 14 (4) | 30 (6.8) | 24 (3.4) | 4 (4.6) | N/A | 0.02 |  |

Supplemental Table 5. Characteristics of participants by joint trajectories in walking speed and ALM/ht^2^ in older men
